# (*E*)-*N*′-(2-Chloro­benzyl­idene)-1-methyl-4-nitro-1*H*-pyrrole-2-carbohydrazide

**DOI:** 10.1107/S1600536813034119

**Published:** 2013-12-24

**Authors:** Jinglin Wang, Rong He, Zhijuan Xin, Huiling Shen, Cairong Wang

**Affiliations:** a Department of Chemistry, Changzhi University, Changzhi, Shanxi 046011, People’s Republic of China

## Abstract

In the title compound, C_13_H_11_ClN_4_O_3_, the phenyl and pyrrolyl ring are linked by an ac­yl–hydrazone (*R*_2_C=N—N—CO—*R*) group, forming a slightly bent mol­ecule: the dihedral angle subtended by the the phenyl and pyrrolyl rings is 8.46 (12)°. In the crystal, the three-dimensional supra­molecular structure is assembled by N—H⋯O hydrogen bonding. Mol­ecular sheets are formed parallel to (101) in a herringbone arrangement by weak van der Waals inter­actions; weak π–π [centroid–centroid phen­yl–phenyl and pyrrol­yl–pyrrolyl distances of 3.7816 (3) and 3.8946 (2) Å, respectively] inter­actions occur between neighbouring sheets.

## Related literature   

For applications and structures of aroylhydrazones, see: Raja *et al.* (2012 ▶); Wang *et al.* (2014 ▶).
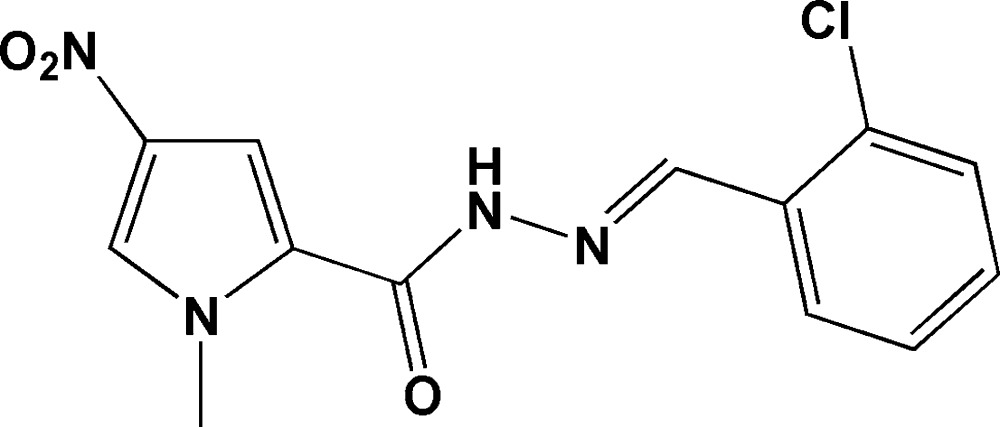


## Experimental   

### 

#### Crystal data  

C_13_H_11_ClN_4_O_3_
*M* *_r_* = 306.71
Monoclinic, 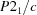

*a* = 13.7649 (13) Å
*b* = 12.4993 (11) Å
*c* = 8.1263 (10) Å
β = 95.523 (1)°
*V* = 1391.7 (2) Å^3^
*Z* = 4
Mo *K*α radiation
μ = 0.29 mm^−1^
*T* = 298 K
0.30 × 0.20 × 0.16 mm

#### Data collection  

Bruker SMART 1000 CCD diffractometer
Absorption correction: multi-scan (*SADABS*; Bruker, 2005 ▶) *T*_min_ = 0.918, *T*_max_ = 0.955
6871 measured reflections
2452 independent reflections
1435 reflections with *I* > 2σ(*I*)
*R*_int_ = 0.071

#### Refinement  

*R*[*F* ^2^ > 2σ(*F* ^2^)] = 0.061
*wR*(*F* ^2^) = 0.164
*S* = 1.00
2452 reflections
191 parameters
H-atom parameters constrained
Δρ_max_ = 0.23 e Å^−3^
Δρ_min_ = −0.33 e Å^−3^

### 

Data collection: *SMART* (Bruker, 1999 ▶); cell refinement: *SAINT* (Bruker, 1999 ▶); data reduction: *SAINT*; program(s) used to solve structure: *SHELXS97* (Sheldrick, 2008 ▶); program(s) used to refine structure: *SHELXL97* (Sheldrick, 2008 ▶); molecular graphics: *DIAMOND* (Brandenburg, 1999 ▶) and *SHELXTL* (Sheldrick, 2008 ▶); software used to prepare material for publication: *PLATON* (Spek, 2009 ▶) and *SHELXTL*.

## Supplementary Material

Crystal structure: contains datablock(s) I. DOI: 10.1107/S1600536813034119/ff2124sup1.cif

Structure factors: contains datablock(s) I. DOI: 10.1107/S1600536813034119/ff2124Isup2.hkl

Supporting information file. DOI: 10.1107/S1600536813034119/ff2124Isup3.cml

Additional supporting information:  crystallographic information; 3D view; checkCIF report

## Figures and Tables

**Table 1:** Hydrogen-bond geometry (Å, °)

*D*—H⋯*A*	*D*—H	H⋯*A*	*D*⋯*A*	*D*—H⋯*A*
N3—H3⋯O1^i^	0.86	2.14	2.941 (3)	154

Symmetry code: (i) 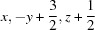.

**Fig. 1.**
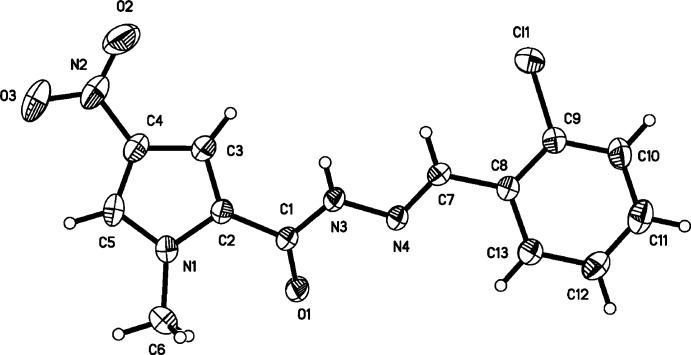
The molecular structure of the title compound with the atom numbering scheme. Displacement ellipsoids are drawn at the 30% probability level. H atoms are presented as small spheres of arbitrary radius.

**Fig. 2.**
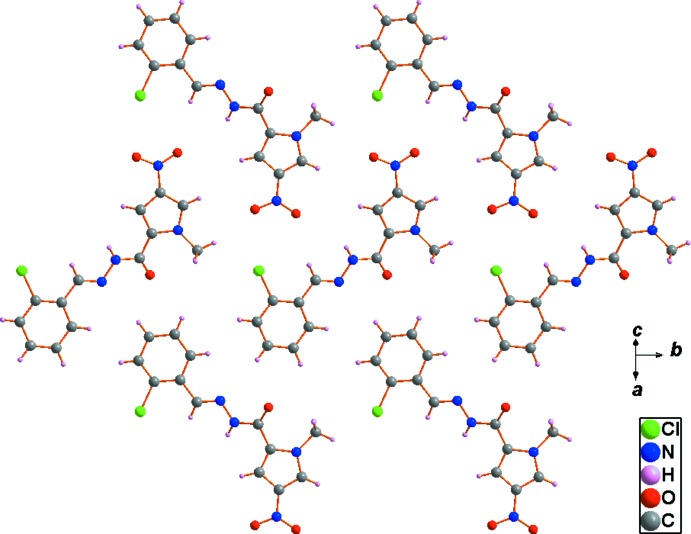
The two-dimensional herringbone layer in the crystal structure of title compound.

**Fig. 3.**
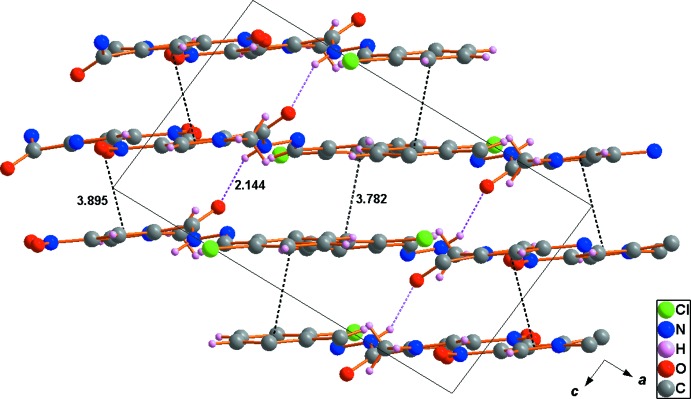
Packing of the title compound viewed along the b axis. The three-dimensional supramolecular structure is assembled by N–H···O hydrogen bonding (pink dotted lines) and weak π···π interactions (black dotted lines) between the neighbouring molecular sheets (all distances in Å).

## supplementary crystallographic information

### 1. Comment

A great number of aroylhydrazones (AH) have triggered wide interest because of their diverse spectra of biological and pharmaceutical properties (Raja, *et al.*, 2012). In our lab, the AH compound (*E*)-*N*'-(2-hydroxybenzylidene)-1-methyl-4-nitro-1*H*-pyrrole-2-carbohydrazide (*L*) and its transition metal complexes were obtained and characterized. The interaction of these compounds with CT-DNA and pBR322 DNA has been explored (Wang, *et al.*, 2014). The present report is an extension of our earlier studies in this area.

In the title compound (Fig. 1), C_13_H_11_ClN_4_O_3_, the phenyl and pyrrolyl ring are linked by acyl-hydrazone (*R*_2_C=N–N–CO–*R*) to form a slightly bent molecule. The dihedral angle between the phenyl (C8—C13) and pyrrolyl rings (C2—C5, N1) is 8.46 (12)°.

As shown in Figure 2, the herringbone molecular sheet of the title compound is formed by weak van-der-Waals interactions along (101) plane.

The three-dimensional supramolecular structure (Fig. 3) is assembled by N3–H3···O1^i^ hydrogen bonding (pink dotted lines) and weak *C*g1···*C*g1^ii^ (*C*g1 is the centroid of the phenyl ring) and *C*g2···*C*g2^iii^ (*C*g2 is the centroid of the pyrrolyl ring) interactions (black dotted lines) between the neighbouring molecular sheets [symmetry code: (i) *x*, 3/2 - *y*, 1/2 + *z*; (ii)1 - *x*, 2 - *y*, 2 – *z*; (iii) - *x*, 1 - *y*, 2 - *z*]. The data of hydrogen-bond geometry are given in Table 1.

### 2. Experimental

Single crystals of the title compound were obtained accidentally in the attempted synthesis of a Ni complex. (*E*)-*N*'-(2-hydroxybenzylidene)-1-methyl-4-nitro-1*H*-pyrrole-2-carbohydrazide (*L*) was synthesized according to literature procedures (Wang *et al.*, 2014). Sodium methoxide (250 µ*L*, 3%, g/V) was added to solution of *L* (0.50 mmol, 0.144 g) in 15 ml MeOH and was heated to reflux. NiCl_2_·6H_2_O (0.50 mmol, 0.119 g) was then added to the refluxing mixture and further refluxed for 2 h. The reaction mixture was cooled and was allowed to stir at room temperature overnight. The mixture was filtered and washed with methanol. The L—Ni complex is not achieved as predicted. However, orange single crystals of the title compound suitable for X-ray analysis were obtained after several days from the mother liquor by slow evaporation.

### 3. Refinement

H atoms attached to C atoms are placed in geometrically idealized position, with N–H=0.86 Å, C–H=0.93 and 0.96 Å, for CH and CH_3_ groups, respectively, and with *U*_iso_(H) = *k* × *U*_eq_(parent C-atom), where *k* = 1.5 for CH_3_ H-atoms and =1.2 for other H-atoms.

### Crystal data

**Table:**

C_13_H_11_ClN_4_O_3_	*F*(000) = 632
*M**_r_* = 306.71	*D*_x_ = 1.464 Mg m^−^^3^
Monoclinic, *P*2_1_/*c*	Mo *K*α radiation, λ = 0.71073 Å
*a* = 13.7649 (13) Å	Cell parameters from 1539 reflections
*b* = 12.4993 (11) Å	θ = 3.0–25.2°
*c* = 8.1263 (10) Å	µ = 0.29 mm^−^^1^
β = 95.523 (1)°	*T* = 298 K
*V* = 1391.7 (2) Å^3^	Block, yellow
*Z* = 4	0.30 × 0.20 × 0.16 mm

### Data collection

**Table:**

Bruker SMART 1000 CCD diffractometer	2452 independent reflections
Radiation source: fine-focus sealed tube	1435 reflections with *I* > 2σ(*I*)
Graphite monochromator	*R*_int_ = 0.071
phi and ω scans	θ_max_ = 25.0°, θ_min_ = 1.5°
Absorption correction: multi-scan (*SADABS*; Bruker, 2005)	*h* = −15→16
*T*_min_ = 0.918, *T*_max_ = 0.955	*k* = −14→14
6871 measured reflections	*l* = −6→9

### Refinement

**Table:**

Refinement on *F*^2^	Primary atom site location: structure-invariant direct methods
Least-squares matrix: full	Secondary atom site location: difference Fourier map
*R*[*F*^2^ > 2σ(*F*^2^)] = 0.061	Hydrogen site location: inferred from neighbouring sites
*wR*(*F*^2^) = 0.164	H-atom parameters constrained
*S* = 1.00	*w* = 1/[σ^2^(*F*_o_^2^) + (0.0787*P*)^2^] where *P* = (*F*_o_^2^ + 2*F*_c_^2^)/3
2452 reflections	(Δ/σ)_max_ = 0.001
191 parameters	Δρ_max_ = 0.23 e Å^−^^3^
0 restraints	Δρ_min_ = −0.33 e Å^−^^3^

### Special details

**Table:**

Geometry. All e.s.d.'s (except the e.s.d. in the dihedral angle between two l.s. planes) are estimated using the full covariance matrix. The cell e.s.d.'s are taken into account individually in the estimation of e.s.d.'s in distances, angles and torsion angles; correlations between e.s.d.'s in cell parameters are only used when they are defined by crystal symmetry. An approximate (isotropic) treatment of cell e.s.d.'s is used for estimating e.s.d.'s involving l.s. planes.
Refinement. Refinement of *F*^2^ against ALL reflections. The weighted *R*-factor *wR* and goodness of fit *S* are based on *F*^2^, conventional *R*-factors *R* are based on *F*, with *F* set to zero for negative *F*^2^. The threshold expression of *F*^2^ > σ(*F*^2^) is used only for calculating *R*-factors(gt) *etc*. and is not relevant to the choice of reflections for refinement. *R*-factors based on *F*^2^ are statistically about twice as large as those based on *F*, and *R*- factors based on ALL data will be even larger.

### Fractional atomic coordinates and isotropic or equivalent isotropic displacement parameters (Å^2^)

**Table:**

	*x*	*y*	*z*	*U*_iso_*/*U*_eq_	
Cl1	0.29032 (9)	1.16858 (8)	1.00653 (18)	0.1044 (6)	
N1	0.14340 (19)	0.51021 (19)	1.0868 (3)	0.0508 (7)	
N2	−0.0430 (2)	0.5940 (3)	1.3388 (4)	0.0712 (9)	
N3	0.24317 (19)	0.7725 (2)	1.0085 (3)	0.0512 (8)	
H3	0.2231	0.7996	1.0964	0.061*	
N4	0.30201 (18)	0.83156 (19)	0.9133 (3)	0.0470 (7)	
O1	0.24317 (17)	0.62828 (16)	0.8366 (3)	0.0587 (7)	
O2	−0.0656 (2)	0.6852 (3)	1.3780 (4)	0.1057 (12)	
O3	−0.08399 (19)	0.5122 (3)	1.3789 (3)	0.0907 (9)	
C1	0.2175 (2)	0.6716 (2)	0.9618 (4)	0.0465 (8)	
C2	0.1506 (2)	0.6203 (2)	1.0708 (4)	0.0451 (8)	
C3	0.0829 (2)	0.6674 (3)	1.1612 (4)	0.0514 (8)	
H3A	0.0711	0.7403	1.1715	0.062*	
C4	0.0352 (2)	0.5839 (3)	1.2344 (4)	0.0561 (9)	
C5	0.0737 (2)	0.4884 (3)	1.1897 (4)	0.0573 (9)	
H5	0.0554	0.4209	1.2235	0.069*	
C6	0.2046 (3)	0.4285 (3)	1.0169 (5)	0.0659 (10)	
H6A	0.2708	0.4362	1.0642	0.099*	
H6B	0.2017	0.4376	0.8992	0.099*	
H6C	0.1810	0.3586	1.0416	0.099*	
C7	0.3117 (2)	0.9297 (2)	0.9548 (4)	0.0483 (8)	
H7	0.2764	0.9569	1.0373	0.058*	
C8	0.3779 (2)	1.0000 (2)	0.8743 (4)	0.0467 (8)	
C9	0.3778 (2)	1.1102 (2)	0.8944 (4)	0.0571 (9)	
C10	0.4438 (3)	1.1761 (3)	0.8256 (5)	0.0698 (11)	
H10	0.4413	1.2498	0.8406	0.084*	
C11	0.5133 (3)	1.1319 (3)	0.7348 (5)	0.0698 (11)	
H11	0.5587	1.1756	0.6900	0.084*	
C12	0.5154 (3)	1.0231 (3)	0.7108 (5)	0.0659 (10)	
H12	0.5620	0.9933	0.6491	0.079*	
C13	0.4481 (2)	0.9577 (3)	0.7784 (4)	0.0568 (9)	
H13	0.4497	0.8843	0.7598	0.068*	

### Atomic displacement parameters (Å^2^)

**Table:**

	*U*^11^	*U*^22^	*U*^33^	*U*^12^	*U*^13^	*U*^23^
Cl1	0.1002 (9)	0.0517 (6)	0.1730 (14)	0.0028 (6)	0.0731 (9)	−0.0179 (7)
N1	0.0553 (16)	0.0427 (14)	0.0560 (18)	−0.0065 (13)	0.0130 (13)	0.0045 (13)
N2	0.057 (2)	0.102 (3)	0.057 (2)	−0.019 (2)	0.0189 (16)	0.0023 (19)
N3	0.0646 (18)	0.0459 (15)	0.0477 (17)	−0.0109 (13)	0.0286 (14)	−0.0042 (12)
N4	0.0549 (16)	0.0436 (15)	0.0455 (16)	−0.0057 (13)	0.0199 (13)	0.0025 (11)
O1	0.0821 (17)	0.0499 (12)	0.0490 (14)	−0.0105 (12)	0.0310 (12)	−0.0064 (11)
O2	0.088 (2)	0.121 (3)	0.117 (3)	−0.010 (2)	0.056 (2)	−0.027 (2)
O3	0.0637 (17)	0.134 (3)	0.077 (2)	−0.0337 (18)	0.0193 (14)	0.0198 (18)
C1	0.053 (2)	0.0444 (17)	0.045 (2)	−0.0027 (15)	0.0197 (15)	0.0023 (14)
C2	0.0476 (18)	0.0425 (16)	0.047 (2)	−0.0063 (14)	0.0123 (15)	−0.0010 (14)
C3	0.0496 (19)	0.0530 (18)	0.054 (2)	−0.0032 (16)	0.0153 (16)	−0.0007 (16)
C4	0.0516 (19)	0.069 (2)	0.050 (2)	−0.0095 (18)	0.0160 (16)	0.0016 (17)
C5	0.059 (2)	0.059 (2)	0.055 (2)	−0.0168 (18)	0.0113 (17)	0.0151 (17)
C6	0.075 (3)	0.0447 (18)	0.079 (3)	0.0036 (18)	0.014 (2)	0.0010 (18)
C7	0.0515 (19)	0.0442 (18)	0.053 (2)	−0.0001 (15)	0.0227 (16)	−0.0005 (15)
C8	0.0473 (18)	0.0431 (16)	0.052 (2)	−0.0039 (15)	0.0150 (15)	0.0006 (14)
C9	0.056 (2)	0.0442 (18)	0.074 (3)	−0.0032 (16)	0.0199 (18)	0.0008 (17)
C10	0.068 (2)	0.0459 (19)	0.098 (3)	−0.0110 (18)	0.021 (2)	0.0092 (19)
C11	0.061 (2)	0.076 (3)	0.074 (3)	−0.013 (2)	0.016 (2)	0.018 (2)
C12	0.063 (2)	0.075 (3)	0.064 (3)	−0.002 (2)	0.0284 (19)	0.003 (2)
C13	0.064 (2)	0.0502 (19)	0.059 (2)	−0.0029 (17)	0.0220 (18)	0.0008 (16)

### Geometric parameters (Å, º)

**Table:**

Cl1—C9	1.739 (3)	C5—H5	0.9300
N1—C5	1.359 (4)	C6—H6A	0.9600
N1—C2	1.387 (4)	C6—H6B	0.9600
N1—C6	1.472 (4)	C6—H6C	0.9600
N2—O3	1.227 (4)	C7—C8	1.465 (4)
N2—O2	1.232 (4)	C7—H7	0.9300
N2—C4	1.439 (4)	C8—C9	1.387 (4)
N3—C1	1.354 (4)	C8—C13	1.402 (4)
N3—N4	1.386 (3)	C9—C10	1.383 (5)
N3—H3	0.8600	C10—C11	1.379 (5)
N4—C7	1.276 (4)	C10—H10	0.9300
O1—C1	1.234 (3)	C11—C12	1.374 (5)
C1—C2	1.483 (4)	C11—H11	0.9300
C2—C3	1.374 (4)	C12—C13	1.388 (4)
C3—C4	1.397 (4)	C12—H12	0.9300
C3—H3A	0.9300	C13—H13	0.9300
C4—C5	1.369 (5)		
			
C5—N1—C2	108.5 (3)	N1—C6—H6B	109.5
C5—N1—C6	124.2 (3)	H6A—C6—H6B	109.5
C2—N1—C6	127.1 (2)	N1—C6—H6C	109.5
O3—N2—O2	124.7 (3)	H6A—C6—H6C	109.5
O3—N2—C4	118.2 (4)	H6B—C6—H6C	109.5
O2—N2—C4	117.1 (3)	N4—C7—C8	120.8 (3)
C1—N3—N4	119.4 (2)	N4—C7—H7	119.6
C1—N3—H3	120.3	C8—C7—H7	119.6
N4—N3—H3	120.3	C9—C8—C13	116.7 (3)
C7—N4—N3	114.6 (2)	C9—C8—C7	122.4 (3)
O1—C1—N3	123.5 (3)	C13—C8—C7	120.9 (3)
O1—C1—C2	123.1 (3)	C10—C9—C8	122.4 (3)
N3—C1—C2	113.3 (3)	C10—C9—Cl1	118.5 (3)
C3—C2—N1	108.5 (3)	C8—C9—Cl1	119.1 (2)
C3—C2—C1	128.8 (3)	C11—C10—C9	119.6 (3)
N1—C2—C1	122.6 (3)	C11—C10—H10	120.2
C2—C3—C4	106.1 (3)	C9—C10—H10	120.2
C2—C3—H3A	126.9	C12—C11—C10	119.8 (3)
C4—C3—H3A	126.9	C12—C11—H11	120.1
C5—C4—C3	109.2 (3)	C10—C11—H11	120.1
C5—C4—N2	124.3 (3)	C11—C12—C13	120.2 (3)
C3—C4—N2	126.4 (3)	C11—C12—H12	119.9
N1—C5—C4	107.6 (3)	C13—C12—H12	119.9
N1—C5—H5	126.2	C12—C13—C8	121.3 (3)
C4—C5—H5	126.2	C12—C13—H13	119.4
N1—C6—H6A	109.5	C8—C13—H13	119.4
			
C1—N3—N4—C7	171.3 (3)	C2—N1—C5—C4	1.7 (4)
N4—N3—C1—O1	−0.5 (5)	C6—N1—C5—C4	177.5 (3)
N4—N3—C1—C2	−177.2 (3)	C3—C4—C5—N1	−1.2 (4)
C5—N1—C2—C3	−1.6 (4)	N2—C4—C5—N1	178.0 (3)
C6—N1—C2—C3	−177.2 (3)	N3—N4—C7—C8	175.1 (3)
C5—N1—C2—C1	−177.7 (3)	N4—C7—C8—C9	168.3 (3)
C6—N1—C2—C1	6.7 (5)	N4—C7—C8—C13	−14.7 (5)
O1—C1—C2—C3	−147.4 (4)	C13—C8—C9—C10	−0.6 (6)
N3—C1—C2—C3	29.3 (5)	C7—C8—C9—C10	176.4 (4)
O1—C1—C2—N1	27.9 (5)	C13—C8—C9—Cl1	178.0 (3)
N3—C1—C2—N1	−155.5 (3)	C7—C8—C9—Cl1	−4.9 (5)
N1—C2—C3—C4	0.8 (4)	C8—C9—C10—C11	−0.7 (6)
C1—C2—C3—C4	176.6 (3)	Cl1—C9—C10—C11	−179.4 (3)
C2—C3—C4—C5	0.3 (4)	C9—C10—C11—C12	1.2 (6)
C2—C3—C4—N2	−178.9 (3)	C10—C11—C12—C13	−0.4 (6)
O3—N2—C4—C5	−6.7 (6)	C11—C12—C13—C8	−1.0 (6)
O2—N2—C4—C5	175.1 (4)	C9—C8—C13—C12	1.5 (5)
O3—N2—C4—C3	172.3 (3)	C7—C8—C13—C12	−175.6 (3)
O2—N2—C4—C3	−5.9 (6)		

### Hydrogen-bond geometry (Å, º)

**Table:**

*D*—H···*A*	*D*—H	H···*A*	*D*···*A*	*D*—H···*A*
N3—H3···O1^i^	0.86	2.14	2.941 (3)	154

Symmetry code: (i) *x*, −*y*+3/2, *z*+1/2.
